# Inhibitory immune checkpoints in preeclampsia: current landscape, mechanisms, and clinical perspectives

**DOI:** 10.3389/fimmu.2026.1940260

**Published:** 2026-08-28

**Authors:** Mi Liu, Guikai Duan, Xiaomin Lai, Bingying Lin, Xiang Li, Jie Yang, Shu Cao, Hao Chen, Tong Wang, Bingyan Chen, Zhihua He, Qinglin Ma, Chun Duan, Yan Zhu, Yuanbin Lu, Yuchi Gao

**Affiliations:** 1 Department of Clinical Laboratory, Shenzhen Maternity and Child Healthcare Hospital, Women and Children’s Medical Center, Southern Medical University, Shenzhen, Guangdong, China; 2 Department of Clinical Laboratory, Shenzhen Third Children's Hospital, Shenzhen, Guangdong, China; 3 Department of Clinical Laboratory, Shenzhen Bao’an District Songgang People’s Hospital, Shenzhen, Guangdong, China; 4 Shenzhen Bao’an Medical College of Guangdong Pharmaceutical University, Shenzhen, Guangdong, China; 5 Shenzhen Key Laboratory of Maternal and Child Health and Diseases, Shenzhen, Guangdong, China

**Keywords:** immune checkpoint, preeclampsia, immune tolerance, maternal-fetal interface, biomarker, immunotherapy

## Abstract

Preeclampsia (PE) is a pregnancy-specific disorder whose pathogenesis remains incompletely elucidated. The prevailing theory suggests that compromised maternal-fetal immune tolerance is a key contributor. In recent years, inhibitory immune checkpoints have garnered considerable attention as key regulatory factors in maintaining immune homeostasis during pregnancy. Programmed cell death protein 1 (PD-1), T cell immunoglobulin and mucin domain-containing protein 3 (Tim-3), cytotoxic T-lymphocyte-associated protein 4 (CTLA-4), T cell immunoreceptor with Ig and ITIM domains (TIGIT), and lymphocyte-activation gene 3 (LAG-3) are well-established inhibitory immune checkpoints in reproductive immunology. Additionally, emerging co-inhibitory molecules such as B- and T-lymphocyte attenuator (BTLA) and V-domain Ig suppressor of T cell activation (VISTA) have been increasingly implicated in the regulation of maternal-fetal immune tolerance. This review focuses on dissecting the mechanisms of inhibitory immune checkpoints in preeclampsia as well as their functions in maternal-fetal immune regulation. We evaluate their potential as early diagnostic biomarkers and novel targets for immunotherapy, highlight the limitations of existing relevant investigations, and outline critical pathways for subsequent clinical translation, offering insights into precision prophylaxis and treatment of preeclampsia.

## 1 Introduction

The maternal immune system must actively tolerate semi-allogeneic fetal antigens throughout gestation, and the disrupted immune regulatory networks underpin the onset and progression of preeclampsia (1). According to international guidelines for hypertensive disorders of pregnancy, preeclampsia is a pregnancy-specific multisystem disorder that occurs exclusively during gestation and the immediate postpartum period. It is defined by *de novo* hypertension (systolic blood pressure ≥140 mmHg and/or diastolic blood pressure ≥90 mmHg, confirmed by two measurements at least 4 hours apart) at or after 20 weeks’ gestation, accompanied by one or more of the following new-onset conditions: proteinuria (≥0.3 g/24 h or a spot urine protein/creatinine ratio ≥30 mg/mmol), maternal end-organ dysfunction, or uteroplacental insufficiency (2, 3). Preeclampsia predisposes patients to life-threatening systemic complications, potentially resulting in maternal or perinatal mortality (4). Based on the gestational age at onset, preeclampsia is clinically classified into early-onset preeclampsia (EOPE) and late-onset preeclampsia (LOPE), with 34 weeks’ gestation used as the threshold (5). Notably, EOPE and LOPE exhibit distinct defects in placental immune homeostasis, laying a critical foundation for exploring differential immune checkpoint alterations across disease subtypes (6). The pathogenesis of preeclampsia remains incompletely understood. It is widely recognized as a progressive, multifactorial disorder involving complex interactions among genetic, environmental, immunological, endocrine, and metabolic factors (7, 8). The current paradigm posits that the disease originates from defective placentation, which subsequently triggers widespread maternal systemic vascular endothelial injury (9). Immune factors play a particularly critical role, as the pathogenesis of preeclampsia is closely associated with immune tolerance imbalance at the maternal-fetal interface and systemic inflammatory alterations, including dysregulation of helper T-cell proportions, polarization toward a pro-inflammatory phenotype macrophages, enhanced decidual natural killer (dNK) cells cytotoxicity, and dysfunctional immune cell-derived cytokine networks (10, 11). Therefore, investigating the immunological changes in preeclampsia is pivotal to elucidating its pathogenic mechanisms.

Immune checkpoints are critical molecules that maintain immune homeostasis. Functionally, they are broadly classified as co-stimulatory and co-inhibitory checkpoints. The inhibitory subset transmits negative regulatory signals to curb excessive autoimmune responses and preserve long-term immune tolerance (12). In preeclampsia, our understanding of classical inhibitory checkpoints (PD-1, Tim-3, CTLA-4, TIGIT, and LAG-3) has advanced considerably, laying a theoretical foundation for clinical translation (13, 14). Concurrently, next-generation co-inhibitory molecules represented by BTLA and VISTA are emerging as research focal points in reproductive immunology (15, 16). Although the roles of these molecules in gestational immune regulation are becoming increasingly recognized, their specific mechanisms of action and targeted intervention strategies in preeclampsia remain to be further elucidated. This review summarizes the structural and functional characteristics of key inhibitory immune checkpoints (**Table 1**), elaborates on the mechanistic roles of classical co-inhibitory checkpoints in preeclampsia, and integrates critical insights from emerging co-inhibitory molecules. The aim is to deepen the understanding of the immunopathological mechanisms underlying preeclampsia and provide a theoretical foundation for developing novel immunodiagnostic biomarkers and targeted therapeutic strategies.

**Table 1:** Key molecular structures and expression characteristics of inhibitory immune checkpoints.

Checkpoints	Alternate names	Ligands	Expression patterns	Signaling motifs	Functions	References
PD-1	CD279	PD-L1 PD-L2	Activated T cells, NKT cells, APCs	ITIM ITSM	Suppresses activation and proliferation of T effectors; Modulate macrophage polarization; Regulates trophoblast behavior.	(17–27)
Tim-3	HAVCR2	Gal-9 CEACAM1 PtdSer HMGB1	T cells, NK cells, DCs, macrophages	Tyrosine-based motif	Induces Th1/Th17 cell apoptosis; Inhibits proliferation and function of NK or T effectors; Enhances function of Tregs/MDSCs.	(28–39)
CTLA-4	CD152	B7-1 (CD80) B7-2 (CD86)	CD4^+^, CD8^+^ T cells, Tregs	YVKM	Binds CD80/86 to competitively block CD28 co-stimulation and induces IDO via reverse signaling.	(40–44)
TIGIT	VSIG9 VSTM3 WUCAM	CD155 CD112	T cells, NK cells, NKT cells	ITIM ITT-like	Binds to CD155/CD112 and inhibits functions of T effectors; Reduces NK cells cytotoxicity and cytokine secretion.	(45–51)
LAG-3	CD223	MHC-II Gal-3 FGL-1	Activated T cells, NK cells, B cells, pDCs	KIEELE	Suppresses T cells function and enhances the suppressive function of Tregs.	(52–60)
BTLA	CD272	HVEM	B cells, DCs, activated T cells, NK cells, macrophages	ITIM ITSM	Suppresses the activation and proliferation of effector T cells; Inhibits cytokine production.	(61–69)
VISTA	PD-1H B7-H5	PSGL-1	Monocytes, macrophages, DCs, CD4^+^ T cells, Tregs	PxxP YxxQ	Inhibits T effectors function and cytokine production; Promotes Tregs differentiation.	(70–72)

## 2 Mechanistic landscape of inhibitory immune checkpoints in preeclampsia

### 2.1 Multidimensional regulatory role of PD-1/PD-L1 axis in preeclampsia

**Figure 1:**
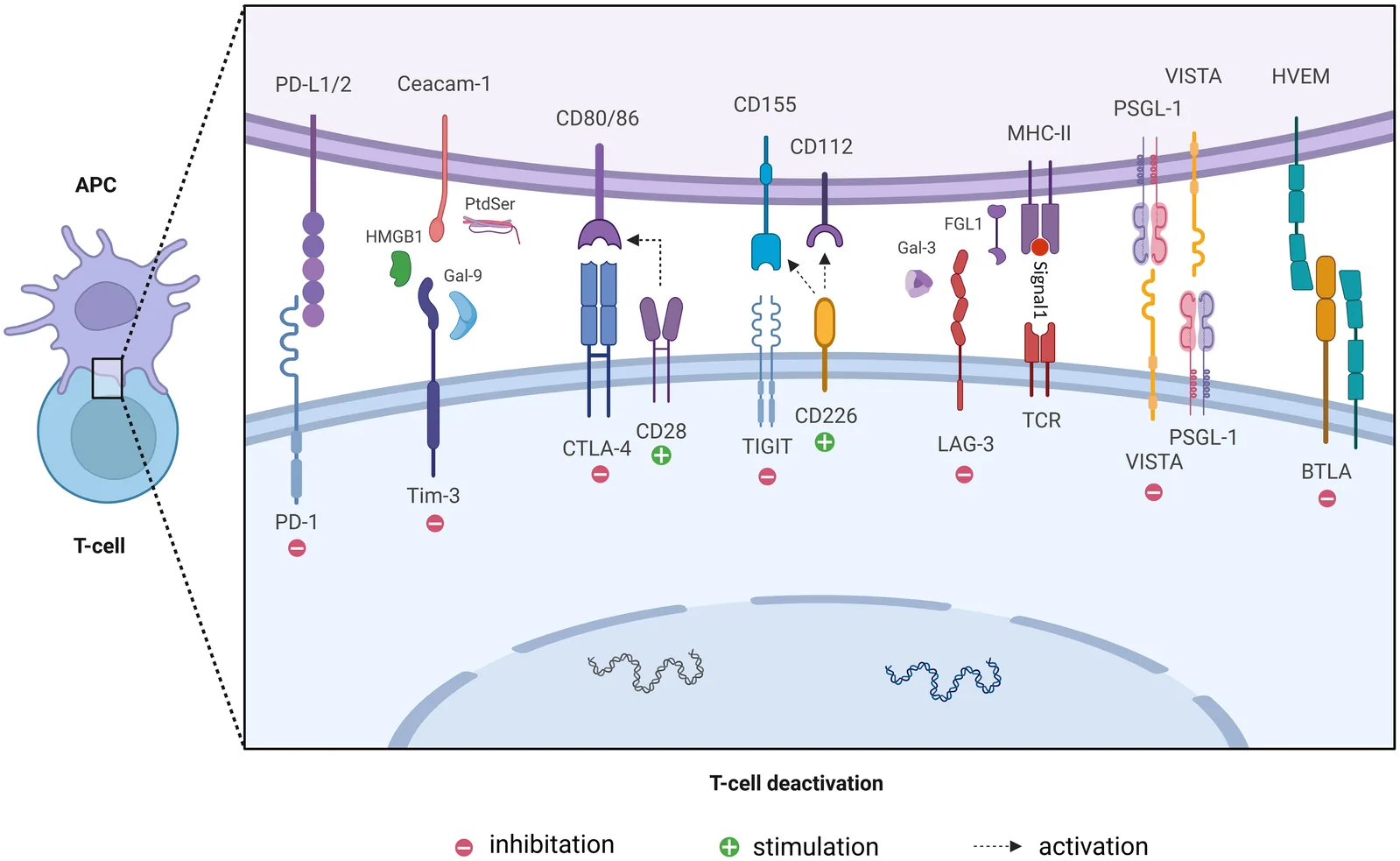
PD-1 binds PD-L1, recruiting SHP-2 via its ITIM/ITSM motifs to inhibit TCR signaling and T cell activation. CTLA-4 outcompetes CD28 for CD80/CD86 via transendocytosis to attenuate co-stimulation, and engages B7 to induce IDO expression for immunosuppression. Tim-3/Gal-9 induces Th1/Th17 apoptosis, suppresses dNK cytotoxicity, and promotes spiral artery remodeling for maternal-fetal tolerance. TIGIT competes with co-stimulatory receptor CD226 for CD155/CD112, downregulating T cell and NK cell functions. LAG-3 inhibits T cells by binding MHC-II with higher affinity than CD4 and via interactions with FGL1/Gal-3, while dimerization also disrupts TCR-MHC-II spacing. BTLA engages HVEM, triggering either SHP-1/SHP-2-mediated inhibition or PI3K/Akt activation, reflecting its bidirectional function. VISTA acts as both receptor and ligand, binding PSGL-1 under acidic conditions to suppress T cell proliferation and cytokine production. Created in BioRender. Liu, M. (2026) https://BioRender.com/me4z58z.

PD-1 is a type I transmembrane glycoprotein composed of an extracellular IgV domain, a transmembrane region, and a cytoplasmic tail containing immunoreceptor tyrosine-based motifs, including immunoreceptor tyrosine-based inhibitory (ITIM) and immunoreceptor tyrosine-based switch (ITSM) motifs (17, 18). PD-1 is primarily expressed on activated T cells, natural killer T (NKT) cells and antigen-presenting cells (APCs) (19, 20). Its primary cognate ligand, PD-L1 (**Figure 1**), is widely distributed, with expression not only on immune cells but also prominently on placental decidual cells (pDCs) and syncytiotrophoblasts (21). Binding of PD-1 to PD-L1 induces phosphorylation of the ITIM and ITSM motifs, recruiting the phosphatase SHP-2 (22, 23). This leads to the dephosphorylation of downstream T cell receptor (TCR) signaling, thereby inhibiting T-cell activation, proliferation, and cytokine production, and sustaining immune tolerance (24). Additionally, this axis modulates decidual macrophage polarization or directly regulates trophoblast behavior, thereby influencing placental function and pregnancy outcomes (25, 26).

During normal pregnancy, PD-1 expression on decidual immune cells and PD-L1 on syncytiotrophoblasts is significantly higher than in the non-pregnant state, creating an immune-privileged microenvironment for the fetus (73, 74). In preeclampsia placentas, dysregulation of the PD-1/PD-L1 axis induces a functional imbalance in T cells, characterized by enhanced pro-inflammatory activity and diminished anti-inflammatory function (75). Specifically, an increase in PD-1-negative or PD-1-low CD8^+^ effector memory T cells (TEM), accompanied by reduction in regulatory T cells (Tregs), impairs maternal-fetal immune suppression (14, 76, 77). Concurrently, downregulated PD-1/PD-L1 axis promotes Th17 cell proliferation, inhibits Treg differentiation, and induces Treg-to-Th17 phenotype conversion, thereby directly disrupting maternal-fetal immune tolerance (75). *In vitro* studies confirm that PD-1/PD-L1 signaling ameliorates the Treg/Th17 imbalance by suppressing the PI3K/AKT/mTOR pathway (75, 78). In decidual tissues of preeclampsia patients, PD-1 downregulation correlates with elevated levels of pro-inflammatory cytokines (IL-1β, IL-6, TNF-α, IFN-γ), and blockade of the PD-1/PD-L1 axis significantly increases certain pro-inflammatory/anti-inflammatory cytokines (IL-1β, IL-6, TNF-α, IFN-γ, IL-4) (79), suggesting PD-1’s role in maintaining dynamic equilibrium within the decidual cytokine network.

The PD-1/PD-L1 axis also modulates innate immune cell functions. Its blockade not only shifts decidual macrophages from an M2 to an M1 phenotype (26, 80), but also impairs their phagocytic capacity, leading to defective clearance of apoptotic cells and exacerbating placental inflammation (81). The expression of PD-1 is found to be significantly downregulated on both decidual macrophages and Hofbauer cells in preeclampsia placentas, while subgroup analyses by fetal sex further reveal that the downregulation of PD-L1 on decidual macrophages is confined exclusively to the male offspring group (82), whereas a recent observation indicates that decidual tissues from male offspring pregnancies harbor a markedly higher proportion of PD-1^HI^Tregs than their female offspring counterparts (83). Collectively, these lines of evidence, despite their divergent functional directions, uniformly point toward a fetal sex-dependent modulation of immune responses via the PD-1/PD-L1 axis, which may in turn shape the immunological milieu at the maternal-fetal interface. These findings indicate that future studies should incorporate fetal sex as a critical covariate. Furthermore, PD-L1 upregulates ARHGDIB via the transcription factor PU.1, thereby enhancing trophoblast migration and invasion (21). In placentas of patients with preeclampsia, PD-L1 sustains GM-CSF expression and promotes trophoblast migration and invasion via the JAK2/STAT5 pathway (84). Overexpression of PD-L1 accelerates the trophoblast cell cycle and enhances their proliferation, whereas blockade of the PD-1/PD-L1 axis suppresses trophoblast migratory and invasive capacities, contributing to the pathogenesis of preeclampsia (21, 84). In subtype analyses, elevated PD-1/PD-L1 expression in peripheral blood of early-onset preeclampsia likely reflects a compensatory response against excessive inflammation (73). However, this compensation may fail to suppress established Th1-dominant immunity once downstream inflammatory cascades are activated (85).

### 2.2 Complex immunoregulatory functions of Tim-3/Gal-9 axis in preeclampsia

Tim-3 is a type I transmembrane glycoprotein. Its extracellular portion consists of an IgV domain and a mucin-like stalk, which are followed by a transmembrane region and a cytoplasmic tail that carries specific tyrosine residues available for phosphorylation (28). Tim-3 is widely expressed on T cells, natural killer (NK) cells, macrophages, and dendritic cells (DCs) (29, 30). Its canonical ligand, galectin-9 (Gal-9) (**Figure 1**), is highly expressed at the maternal-fetal interface, notably on decidual stromal cells, endothelial cells and various immune cells, including T cells, B cells, and macrophages (31). The Tim-3/Gal-9 interaction promotes maternal-fetal immune tolerance by inducing apoptosis of Th1 and Th17 cells, suppressing dNK cell cytotoxicity and their inflammatory cytokine production, while facilitating spiral artery remodeling and pregnancy maintenance (32–35). During normal pregnancy, Tim-3 exhibits differential expression across different gestational stages and immune cell subsets, whereas Gal-9 maintains persistently high expression throughout the entire gestational period (31). In peripheral blood from patients with preeclampsia, Tim-3 is generally downregulated on most immune cell subsets, whereas Gal-9 is elevated (36). Meanwhile, both are increased in preeclampsia decidual tissue compared to normal pregnancies, accompanied by aberrant decidual macrophage polarization, enhanced Th1/Th17 responses, impaired Tregs and myeloid-derived suppressor cell (MDSC) suppression, and increased NK cell cytotoxicity (37, 38).

Decidual macrophages (DMs) play a critical role at the maternal-fetal interface. In preeclampsia, downregulation of Tim-3/Gal-9 signaling promotes M1 macrophage polarization, impairing placental vascular remodeling (86). Exogenous Gal-9 activats this axis to induce M2 polarization of DMs and ameliorate hypertension and proteinuria in preeclampsia-like rat models (87). However, emerging evidence demonstrates that trophoblast-derived Gal-9 activates CD11c-high DMs via CD44 binding, suppressing spiral artery remodeling and contributing to preeclampsia pathogenesis, and exogenous Gal-9 induced preeclampsia-like symptoms in healthy pregnant mice, while Gal-9 blockade prevented these changes (88), which means Gal-9 participates in maternal-fetal immune regulation via Tim-3-independent pathways. This may explain why blocking Tim-3 alone fails to fully recapitulate all phenotypes of Gal-9 deficiency, and highlights a mechanistic framework for understanding the multifaceted role of Gal-9 in preeclampsia. We propose a receptor-switching hypothesis: the function of Gal-9 is context-dependent, governed by receptor availability and the local cytokine milieu. Under conditions of intact Tim-3 signaling, Gal-9 preferentially engages Tim-3 to promote M2 polarization and immune tolerance. However, when Tim-3 is downregulated (as in preeclampsia), Gal-9 may shift to engage alternative receptors such as CD44, activating pro-inflammatory CD11c-high DMs. This receptor-switching hypothesis warrants further investigation.

The Tim-3/Gal-9 pathway promotes Th1 cell apoptosis, downregulating Th1-type immunity, fostering a Th2 shift, and contributes to Treg/Th17 bidirectional regulation (89). Early research reported upregulated Tim-3/Gal-9 with increased IFN-γ and IL-17 and decreased IL-10, skewing the balance toward Th1/Th17 (37). Others reported downregulated Tim-3 in preeclampsia peripheral blood and decidua (13, 90). A recent study has revealed that the expression of Gal-9 and Tim-3 exhibits significant regional heterogeneity across distinct anatomical compartments of the maternal-fetal interface, including the chorionic villi, decidua, and myometrium, with Gal-9 expression being markedly higher in the decidua than in the chorionic villi (31). Another study performed subgroup analyses stratified by fetal sex and found that the downregulation of Gal-9 expression on decidual macrophages and Hofbauer cells was confined exclusively to the male offspring group (38). These observed discrepancies in expression profiles are likely attributable not only to cellular subset heterogeneity or differences in disease subclassification, but also to fetal sex, a covariate that warrants due consideration.

During normal pregnancy, MDSCs accumulate in the placenta, synergizing with Tregs to exert immunosuppressive functions (91, 92). In preeclampsia, MDSCs transmit inhibitory signals by upregulating Tim-3 and Gal-9, thereby counteracting hyperactivated immune responses. Conversely, blocking Tim-3/Gal-9 reduces MDSC-mediated T cell suppression (93). Additionally, Tim-3 serves as an NK cell maturation marker, and its expression is significantly upregulated upon stimulation with cytokines such as IL-12, IL-18 and IL-15, promoting NK cell maturation and acquisition of effector functions (94). Downregulated Tim-3 on peripheral blood NK cells in early-onset preeclampsia allows them to escape Gal-9-mediated suppression, leading to enhanced cytotoxicity and increased Th1 cytokine release, potentially driving the disease pathogenesis (36, 90). Future studies should prioritize single-cell RNA sequencing of decidual vs. peripheral NK cells to elucidate Tim-3-mediated differential regulation.

### 2.3 Dual immunoregulatory mechanism of CTLA-4 and genetic susceptibility

CTLA-4, a CD28 homolog, is a type I transmembrane glycoprotein comprising an extracellular IgV domain (mediating homodimerization), a transmembrane region, and a short cytoplasmic tail with a YVKM motif (40, 41). It is expressed on activated CD4^+^ and CD8^+^ T cells, and is constitutively expressed at high levels on Tregs (42). The immunomodulatory mechanism of CTLA-4 involves dual pathways: (i) removing CD80/CD86 from APC surfaces via transendocytosis, thereby blocking CD28 co-stimulatory signals (**Figure 1**) (43); and (ii) on Tregs, CTLA-4 engagement can transmit a reverse signal through B7 molecules to induce IDO expression in APCs, further contributing to T cell suppression (44). In peripheral blood from women with preeclampsia, significantly reduced levels of both membrane-bound and soluble CTLA-4 lead to excessive activation of the B7/CD28 pathway, exacerbating immune tolerance imbalance and contributing to disease pathogenesis (13).

During normal pregnancy, Tregs in the decidua express higher levels of CTLA-4 than those in peripheral blood (95). In peripheral blood from patients with preeclampsia, B7–1 and B7–2 expression on monocytes and IDO expression in T cells were significantly decreased, indicating that CTLA-4-mediated suppression via B7 reverse signaling is impaired (96). A recent study reported that CTLA-4 expression is significantly downregulated in decidual tissue from patients with preeclampsia (97), while the proportions of CTLA-4^+^ Tregs and CCR4^+^ Tregs in peripheral blood are elevated (98, 99). In addition, elevated serum sCTLA-4 and sCD80 levels have also been reported in preeclampsia (100). Those suggest that Treg homing to the maternal-fetal interface may be impaired in preeclampsia, resulting in insufficient local immunosuppression.

At the genetic level, early studies reported a significant association between the CTLA-4 gene 49A/G polymorphism and preeclampsia (101, 102). However, subsequent studies failed to replicate this conclusion (103, 104). A recent meta-analysis of 12 studies (4,658 participants) found no significant association across any genetic model, with trial sequential analysis confirming insufficient evidence (105). This heterogeneity likely arises from variations in sample size, methodological approaches, and ethnic backgrounds. Therefore, future large-scale, multicenter studies combined with functional experiments are required to further elucidate the specific role of CTLA-4 gene polymorphisms in preeclampsia.

### 2.4 TIGIT: compensatory inhibition and subtype-specific expression in preeclampsia

TIGIT is a type I transmembrane glycoprotein composed of an extracellular IgV domain, a transmembrane region, and an intracellular tail with an ITIM and an immunoglobulin tail tyrosine (ITT-like) motif (45). TIGIT is predominantly expressed on CD4^+^/CD8^+^ T cells, Tregs, NK cells, and NKT cells, with emerging evidence revealing its expression and function in additional types (46). Its extracellular immunoglobulin variable (IgV) domain binds poliovirus receptor (CD155) and nectin-2 (CD112) (**Figure 1**). Upon ligand engagement, the ITT-like motif in its cytoplasmic tail is phosphorylated and recruits the adaptor GRB2, which in turn recruits SHIP1 to mediate inhibitory signaling (47). Binding of TIGIT to CD155/CD112 on APCs downregulates effector T cell function and TCR expression, reduces NK cell cytotoxicity and cytokine secretion, and maintains an immune-tolerant microenvironment (48). The co-stimulatory receptor DNAX accessory molecule 1 (DNAM-1, also known as CD226) competes with TIGIT for CD155 and CD112, but exhibits markedly lower binding affinity (49).

In early-onset preeclampsia peripheral blood, CD226 expression is decreased on NK cells, monocytes and diverse T-cell subpopulations, while monocyte CD155 and CD112 expression is increased, suggesting that the dominant inhibitory signals mediated by TIGIT/CD155 and TIGIT/CD112 attempt to curb excessive inflammatory responses (106). Integrating this with the earlier finding that CD226 is instrumental in sustaining maternal immune tolerance during pregnancy, the failure of this compensatory inhibition, rather than the mere presence of inhibitory signals, may constitute a pivotal determinant in the immunopathogenesis of early-onset preeclampsia (107).

Alterations in TIGIT signaling exhibit cell-subset specificity (46). In the peripheral blood of patients with EOPE, while the surface expression of TIGIT on NKT cell subsets did not differ significantly from that in healthy pregnant controls, the relative CD226 expression was significantly decreased across all investigated NKT subpopulations (CD4^+^, CD8^+^, CD4^+^CD8^+^, CD4^-^CD8^-^) (85). Another study found no significant differences in TIGIT, CD226 or CD155 expression on CD4^+^, CD8^+^ or Treg cells between preeclampsia patients and healthy pregnant women, although Treg percentages were significantly lower in the preeclampsia group (108). Unlike peripheral blood, the expression patterns of TIGIT and its ligands at the maternal-fetal interface are more complex. In EOPE placentas, mRNA levels of TIGIT, CD155, CD112 were upregulated 2-fold compared to control levels, whereas in late-onset preeclampsia, they were only one-third of control levels. However, CD112 and CD155 protein levels were significantly reduced in both preeclampsia subtypes (109). This suggests a compensatory transcriptional upregulation in EOPE that fails to translate into functional protein, leading to immune tolerance failure, while late-onset preeclampsia shows downregulation at both transcriptional and protein levels. These contrasting patterns highlight the critical need for clinical subtype stratification in mechanistic studies of preeclampsia. Future prospective studies examining the dynamic changes of these molecules across subtypes, tissues, and cell subsets are needed to establish causality and therapeutic potential.

### 2.5 LAG-3 in preeclampsia: tissue specificity and subtype-differential expression

LAG-3, a CD4 homolog, is a type I transmembrane glycoprotein. Its extracellular domain consists of four immunoglobulin-like domains (D1-D4), while its cytoplasmic tail contains a conserved KIEELE motif, which is essential for inhibitory function (52, 53). LAG-3 is primarily expressed on activated T cells (including CD4^+^, CD8^+^ T cells and Tregs), NK cells, B cells and pDCs (54, 55). LAG-3 suppresses T cell function via multiple interrelated mechanisms (56). Its D1 domain binds MHC-II with significantly higher affinity than CD4, competitively blocking CD4 engagement, while LAG-3 dimerization may additionally enforce suboptimal TCR-MHC-II spacing that attenuates T cell activation (57, 58). Additionally, LAG-3 engagement by non-MHC-II ligands, particularly fibrinogen-like protein 1 (FGL-1) and potentially galectin-3 (Gal-3) (59), further contributes to T cell inhibition and enhances the suppressive function of Tregs (**Figure 1**) (60).

During normal pregnancy, LAG-3 expression is significantly higher in placental tissue than in peripheral blood, decidual CD4^+^ T cells highly express LAG-3, which coincides with enhanced Treg suppressive activity, suggesting synergy among multiple co-inhibitory receptors at the maternal-fetal interface (110–112). Functional studies have demonstrated that blocking LAG-3/FGL-1 axis promotes T cell proliferation and leading to cytokine (e.g., IL-2) production, thereby contributing to immune hyperactivation and impaired tolerance (59).

In the peripheral blood of patients with EOPE, CD4^+^ and CD8^+^ T cells exhibit reduced LAG-3 expression compared with healthy pregnant women, coupled with significantly elevated plasma FGL-1 levels (113). Furthermore, low LAG-3 expression on CD8^+^ NKT cells may correlate with enhanced cytotoxicity and exacerbated maternal Th1 skewing (85). This imbalanced ligand-receptor axis likely reflects a compensatory attempt to counteract impaired LAG-3 signaling, ultimately failing to restrain Th1-skewed peripheral immune responses. Unlike its expression in peripheral blood, LAG-3 exhibits a unique expression pattern at the maternal-fetal interface (114). LAG-3 expression on decidual T cells is significantly higher than that on peripheral blood T cells in both normal pregnancies and patients with EOPE (113). Despite lower overall LAG-3 levels in EOPE, relatively high expression persists locally in the decidua, suggesting a compensatory protective mechanism that attempts to curb excessive inflammation and slow disease progression (14). Collectively, these findings underscore the essential negative regulatory role of LAG-3 across diverse immune cell types and emphasize the need for tissue-specific strategies for modulating immune responses. Future research should elucidate the dynamic interplay between decidual and systemic LAG-3 signaling and the differential regulation of LAG-3 across preeclampsia subtypes.

## 3 Other pregnancy-associated inhibitory immune checkpoints

### 3.1 BTLA

BTLA is a type I transmembrane glycoprotein comprising an extracellular IgV-like domain, a transmembrane region, and a cytoplasmic tail containing ITIM, ITSM and Grb2-binding motifs (61). BTLA is primarily expressed on B cells and activated T cells, with low expression on macrophages, DCs, and NK cells (62). It possesses dual immunomodulatory potential, mediating both inhibitory and stimulatory functions (63). Its inhibitory ligand is the herpesvirus entry mediator (HVEM) (**Figure 1**). Interaction of BTLA’s Ig domain with HVEM’s cysteine-rich domain1 induces ITIM/ITSM phosphorylation, recruiting SHP-1/SHP-2 for inhibitory signaling (64, 65). However, BTLA’s Grb2 motif can recruit the Grb2-PI3K complex, activating the PI3K/Akt pathway under certain conditions, contributing to immune activation (66). BTLA can also act as a ligand, transmitting activating signals to HVEM-expressing T cells (67). This confers upon BTLA a bidirectional regulatory potential that is unique among inhibitory immune checkpoints.

Current understanding of BTLA in maternal-fetal immune tolerance is largely extrapolated from oncology and infectious disease studies, and direct evidence in preeclampsia is lacking. BTLA function is cell-type- and pathology- dependent. In mouse models of hepatitis B virus (HBV)-induced acute or chronic liver failure, BTLA engagement can trigger the PI3K/Akt pathway, which not only inhibits the activation and proliferation of CD4^+^ T cells but also promotes their apoptosis, accelerating disease progression (115). In active tuberculosis, high BTLA expression on DCs downregulates IL-12 and IFN-α while promoting IL-4 and TGF-β production, thereby inhibiting Th17/Th22 responses and promoting Th2/Treg differentiation, underscoring its immunosuppressive role (116, 117). The role of BTLA in maternal-fetal immune tolerance remains poorly understood. A study reported that overall BTLA expression in peripheral blood or decidua show no difference in women with unexplained recurrent pregnancy loss (URPL) (118). Its contribution to immune imbalance at the maternal-fetal interface and its potential link to preeclampsia warrant further investigation.

### 3.2 VISTA

VISTA is a type I transmembrane glycoprotein comprising an extracellular IgV domain, a stalk region, a transmembrane domain, and a cytoplasmic tail containing a conserved SH2-binding motif but lacking classical ITIM/ITSM motifs (70). VISTA is broadly expressed within the hematopoietic compartment, with highest expression on myeloid cells (monocytes, macrophages, DCs) and moderate expression on CD4^+^ T cells and regulatory T cells (119). Its extracellular IgV domain shares high homology with the PD-L1, while its cytoplasmic tail resembles that of CD28 and CTLA-4. This structural feature allows VISTA to function as both a receptor and a ligand (120). Functioning as an inhibitory checkpoint, VISTA suppresses T cell proliferation and pro-inflammatory cytokines (e.g., IL-2, TNF-α, IFN-γ) production (121). Moreover, *in vitro* studies have shown that VISTA can promote naive T cells into Tregs upon TGF-β stimulation, enhancing immune suppression (122). Notably, under acidic conditions, VISTA exerts its immunosuppressive function by selectively binding to P-selectin glycoprotein ligand 1 (PSGL-1) (**Figure 1**) (71). Given the potentially acidic microenvironment at the maternal-fetal interface, a condition that may be exacerbated in preeclampsia due to placental hypoxia and enhanced glycolysis, VISTA may play a unique regulatory role at this site (15). However, research on VISTA in maternal-fetal immune tolerance remains limited, and its potential involvement in preeclampsia warrants deeper exploration. Specifically, the mechanistic details of VISTA-mediated regulation at the maternal-fetal interface, particularly its interactions with distinct immune cell subsets and its potential crosstalk with other checkpoint pathways, remain poorly understood. Addressing these gaps will be critical for evaluating VISTA as a therapeutic target in preeclampsia.

## 4 Clinical translation prospects of inhibitory immune checkpoints in preeclampsia

The successful clinical translation of inhibitory immune checkpoints, particularly in oncology, coupled with their known mechanisms in autoimmunity, provides a strong rationale for exploring their therapeutic potential in preeclampsia. Several studies highlight their potential as early diagnostic markers for preeclampsia. Compared to normal pregnancies, the expression of PD-1 and Tim-3, along with their respective ligands PD-L1 and Gal-9, on M1 and M2 macrophages is significantly reduced in preeclampsia placentas (26, 38). Given that downregulation of CTLA-4 at the maternal-fetal interface is closely linked to local immune tolerance imbalance in preeclampsia, profiling these molecules at the maternal-fetal interface or in peripheral blood may provide adjunctive indicators for assessing placental immune status and predicting preeclampsia risk (97). To date, however, no studies have reported quantitative diagnostic metrics (e.g., sensitivity, specificity, AUC) for these checkpoint molecules as preeclampsia biomarkers, and this represents a critical gap that hampers clinical translation.

Animal models and *in vitro* experiments have provided proof-of-concept for immune checkpoint-targeted therapy. PD-L1 monoclonal antibody treatment effectively reverses the Treg/Th17 imbalance, improves macrophage polarization, and alleviates preeclampsia-like symptoms (78, 80). PD-L1 overexpression enhances trophoblast migration, invasion, and proliferation while inhibiting apoptosis, functions associated with improved placental development and fetal growth (21, 84). Targeting the Tim-3/Gal-9 axis also shows therapeutic promise (87). Importantly, several studies have reported synergistic effects with combined interventions. Within the unique immunological microenvironment at the maternal-fetal interface, the Tim-3 and CTLA-4 pathways exhibit synergistic effects in modulating the interactions between decidual immune cells (DICs) and extravillous trophoblasts; dual blockade cooperatively downregulates the secretion of IL-4 and IL-10, thereby markedly reversing the DIC-mediated promotion of extravillous trophoblast proliferation, invasion, and vasculogenic capacity (123). Consistently, Wang et al., in two separate studies, observed that concurrent blockade of CTLA-4 and Tim-3 dampens the production of Th2/Treg-type cytokines from decidual CD4^+^ and CD8^+^ T cells while promoting Th1-type inflammatory responses, a shift that directly compromises the immune-protective environment essential for successful pregnancy maintenance (124, 125). These findings suggest that the two pathways may functionally complement each other in sustaining normal pregnancy. In an LPS-induced rat preeclampsia model, combined administration of PD-L1 and Gal-9 fusion proteins provided superior protection compared to either agent alone. Mechanistically, concurrent activation of the PD-1/PD-L1 and Tim-3/Gal-9 pathways, via synergistic engagement of the ERK/GSK3β/β-catenin signaling axis, effectively suppresses M1-type macrophage polarization. This combined approach achieves greater amelioration of preeclampsia-like manifestations, including hypertension and proteinuria, as well as improved placental development, relative to single-pathway intervention (80). Collectively, these findings suggest that multi-target combination strategies may represent a promising direction for preeclampsia immunotherapy.

## 5 Discussion and future perspective

### 5.1 Current challenges and bottlenecks

While current research demonstrates the translational potential of inhibitory immune checkpoints for preeclampsia diagnosis and targeted therapy, several challenges impede clinical application. A major source of concern is the substantial heterogeneity across study design. Populations differ widely by ethnicity and geography, and inconsistencies in tissue sampling, disease subtyping, and gestational age stratification are common. In addition, the role of fetal sex has received insufficient attention in prior studies on immune checkpoint expression at the maternal-fetal interface in preeclampsia. Among the landmark studies included in this review, only a few groups have performed stratified analyses, and their findings suggest differential expression of selected checkpoints and ligands according to fetal sex. This clinical variable may well influence the maternal-fetal immune tolerance microenvironment by modulating immune checkpoint expression profiles at the interface, and thus deserves more systematic consideration in future investigations.

Methodologically, most evidence relies on animal models or *in vitro* systems with limited translatability to human preeclampsia pathophysiology, and the dominance of conventional techniques such as flow cytometry and qPCR means that the majority of findings remain at a correlative level, with insufficient functional validation to support causal inference. Another layer of complexity is that preeclampsia involves the simultaneous dysregulation of multiple co-inhibitory and co-stimulatory molecules across diverse immune cell subsets; yet most studies focus on individual molecules in specific cell subsets, overlooking the network-level interplay that likely governs immune homeostasis at the maternal-fetal interface. This single-molecule approach is particularly problematic given emerging evidence that different checkpoints are capable of both redundant and distinct regulatory functions, depending on the cellular or microenvironmental context. In some settings, pathways operate in parallel to produce additive effects; in others, they converge on shared cascades to reinforce a common functional outcome.

From a clinical translation standpoint, quantitative diagnostic metrics (such as sensitivity, specificity, AUC) are uniformly absent, and no interventional trials have been reported. Safety considerations remain largely unexplored. A theoretical framework can be informed by oncology data, where immune checkpoint inhibitors have been accidentally administered during pregnancy. Although isolated case reports document uneventful outcomes in some exposed patients, the aggregate evidence across PD-1, CTLA-4, Tim-3, and LAG-3 pathways consistently indicates that pharmacological blockade disrupts maternal-fetal immune tolerance, with adverse outcomes including miscarriage, preterm delivery, and fetal growth restriction. While these findings derive from receptor antagonists rather than agonists, they indirectly support the premise that agonistic engagement, by reinforcing inhibitory signals, may theoretically favor immune tolerance. Conversely, excessive immunosuppression could predispose to maternal infections or interfere with fetal immune development. Placental transfer and fetal exposure remain entirely uncharacterized; transplacental passage depends on molecular weight and receptor-binding properties, and no data currently exist regarding whether these molecules reach the fetal circulation. These uncertainties underscore the need for systematic evaluation in developmental toxicity studies and early-phase trials. Collectively, these gaps highlight the urgent need for better-designed, functionally grounded, and clinically oriented studies to move the field forward.

### 5.2 Future directions and translational pathways

To address the above limitations and accelerate clinical translation, future investigations should be strategically oriented toward the following priorities. At the methodological level, adopting multi-omics and high-dimensional approaches (e.g., single-cell sequencing, mass cytometry and spatial profiling technologies) will be essential to deconstruct the dynamic, cell-type-specific interplay of checkpoint molecules across the entire immune landscape (35, 126). Such efforts must be rigorously stratified by disease subtype (EOPE vs. LOPE) and key covariates, such as fetal sex, to construct a network-level understanding of immune dysregulation. To move beyond correlation, future directions should pivot from correlative descriptions toward mechanistic explanations, ideally through functional intervention assays and animal models that recapitulate human-like pathological traits. On the diagnostic front, well-designed prospective cohort studies with predefined biomarker endpoints are urgently needed to formally quantify predictive performance using standardized metrics (such as sensitivity, specificity, and AUC), thereby establishing whether these molecules genuinely function as clinically actionable biomarkers. Critically, the translational roadmap must prioritize safety, a dimension that has remained conspicuously unexplored. Early-phase efforts should prioritize comprehensive toxicology and pharmacokinetic profiling in pregnant animal models, coupled with the parallel development of targeted delivery strategies to mitigate off-target fetal exposure. Only upon robust preclinical target validation—grounded in functional intervention assays and pathogenesis-relevant animal models—can first-in-human interventional trials be rationally designed, with rigorous ethical oversight and predefined maternal-fetal outcome measures.
